# Whole-genome sequencing of two multidrug-resistant acinetobacter baumannii strains isolated from a neonatal intensive care unit in Egypt: a prospective cross-sectional study

**DOI:** 10.1186/s12866-024-03482-3

**Published:** 2024-09-21

**Authors:** Rania Alam Eldin Mohamed, Nouran Magdy Moustafa, Fatma Mostafa Mahmoud, Yara Said Elsaadawy, Heba Sherif Abdel Aziz, Shaimaa Abou Bakr Gaber, Abdelrahman Mohamed Hussin, Mohamed G. Seadawy

**Affiliations:** 1 Medical Microbiology and Immunology Department, Faculty of Medicine, Ain Shams University, Cairo, Egypt; 2 Basic Medical Science Department, College of Medicine, Dar Al Uloom University, Riyadh, Saudi Arabia; 3 Clinical and Chemical Pathology Department, Faculty of Medicine, Cairo University, Cairo, Egypt; 4 Clinical Pathology Department, Faculty of Medicine, Ain Shams University, Cairo, Egypt; 5 Armed Forces College of Medicine, Cairo, Egypt; 6 Biodefense Center for Infectious and Emerging Diseases, Ministry of Defense, Cairo, Egypt

**Keywords:** Multidrug-resistant *a. baumannii*, Antibiotic resistance genes, Whole-genome sequencing, Multilocus sequence typing

## Abstract

**Background:**

*Acinetobacter baumannii* (*A. baumannii*) is a life-threatening and challenging pathogen. In addition, it accounts for numerous serious infections, particularly among immunocompromised patients. Resistance to nearly all clinically used antibiotics and their ability to spread this resistance is one of the most important concerns related to this bacterium.

**Objectives:**

This study describes different molecular mechanisms of two multidrug-resistant *A. baumannii* isolates obtained from endotracheal aspirates collected from the neonatal intensive care unit (NICU), Ain Shams University Hospital, Egypt.

**Methods:**

Following the identification of two isolates, they were examined for susceptibility to antimicrobial agents. This was followed by multilocus sequence typing as well as whole-genome sequence (WGS). Additionally, a Pathosystems Resources Integration Center (PATRIC) analysis was performed.

**Results:**

Two isolates, Ab119 and Ab123, exhibited resistance to all tested antibiotics except for tigecycline and colistin. The WGS analysis of antimicrobial resistance genes (AMR) indicated that both isolates shared beta-lactam*,* aminoglycoside*,* macrolides, and sulfonamide resistance genes. Furthermore, each strain revealed different resistance genes such as *blaNDM-1, blaNDM-10, OXA-64, aph (3')-VI, Tet-B* in Ab119 strain and *blaOXA-68, blaPER-1, blaPER-7, Tet-39* in Ab123 strain. Multiple efflux pump genes were detected. Multilocus sequence typing indicated that both isolates belong to the same sequence type (ST931), which belongs to international clone (IC3). Both isolates exhibited the presence of multiple mobile genetic elements (MGEs), but no plasmid was detected in either of them.

**Conclusions:**

A low prevalence of the IC3 sequence type was identified among two *A. baumannii* isolates obtained from the NICU in Egypt, exhibiting a high resistance level. Healthcare workers must have knowledge regarding the prevalence of *A. baumannii* among different populations in order to administer suitable treatment, improve patient outcomes, and apply effective infection control practices.

**Supplementary Information:**

The online version contains supplementary material available at 10.1186/s12866-024-03482-3.

## Background

*Acinetobacter baumannii* (*A. baumannii*) is generally a non-pathogenic microorganism. Nevertheless, in the past forty years, it has been discovered to be a severe pathogen in hospitals [1]. Typically, it is associated with hospital-acquired infections like urinary tract infections, bacteremia, lower respiratory tract infections, meningitis, and wound infections [2–5]. Community-acquired infections induced by *A. baumannii* have been identified, particularly in individuals with comorbidities [6–8]. Neonatal infections caused by *A. baumannii* are increasing, with a corresponding increase in the frequency of their isolation. In addition, fatality rates due to these infections are more than fatality rates caused by other isolated organisms [9]. Neonates who are born prematurely, have low birth weight, use invasive devices like endotracheal intubation and intravascular catheterization, receive parenteral nutrition, and undergo broad-spectrum antibiotic therapy are more likely to acquire *A. baumannii* infections [10].

*A. baumannii* is a significant concern in healthcare facilities globally because it has the capacity to develop and gain resistance to nearly all antibiotics utilized [11]. This risk is significantly amplified amongst patients in intensive care units (ICUs), where death rates can increase up to 40% [12]. *A. baumannii* has experienced a rapid emergence of antibiotic-resistant strains on a global scale. *A. baumannii* typically acquires intrinsic resistance by reducing membrane permeability, producing various types of ß-lactamase enzymes, and exhibiting efflux pump activity [13]. The presence of AMR in *A. baumannii* is typically associated with MGEs that can be transferred between bacteria, facilitating the rapid spread as well as retention of resistance genes across varying bacterial species [14]. Resistance can also be obtained through mutational alterations in the structure of chromosomes, the horizontal transfer of genes [15], and some naturally present intrinsic resistance genes [16].

*A. baumannii* possesses a remarkable ability to develop AMR from different sources, disseminate it, and evolve novel mechanisms of resistance [17]. Moreover, it can quickly develop extrinsic resistance mechanisms throughout treatment by obtaining additional genetic traits (via cross-species horizontal gene transfer) [18, 19]. The *A. baumannii* genome comprises a chromosome and numerous plasmids primarily associated with developing AMR genes [20]. *A. baumannii* strains’ comparative genomic analysis indicated that *A. baumannii* genome has the ability to incorporate a significant amount of DNA from external sources. This process may contribute to the development of AMR and pathogenesis [21, 22].

Therefore, this study aims to explore, by WGS, different antibiotic resistance mechanisms of *A. baumannii* strains (isolated from the NICU) at Ain Shams University (ASU) Hospital.

## Methods

### Ethical approval

The Research Ethical Committee, Faculty of Medicine, Ain Shams University, granted approval for this study under the code No: FMASU R02/2024. Informed consent to participate was obtained from all the legal guardians of the patients.

### Settings, study design and isolates selection

**Fig. 1:**
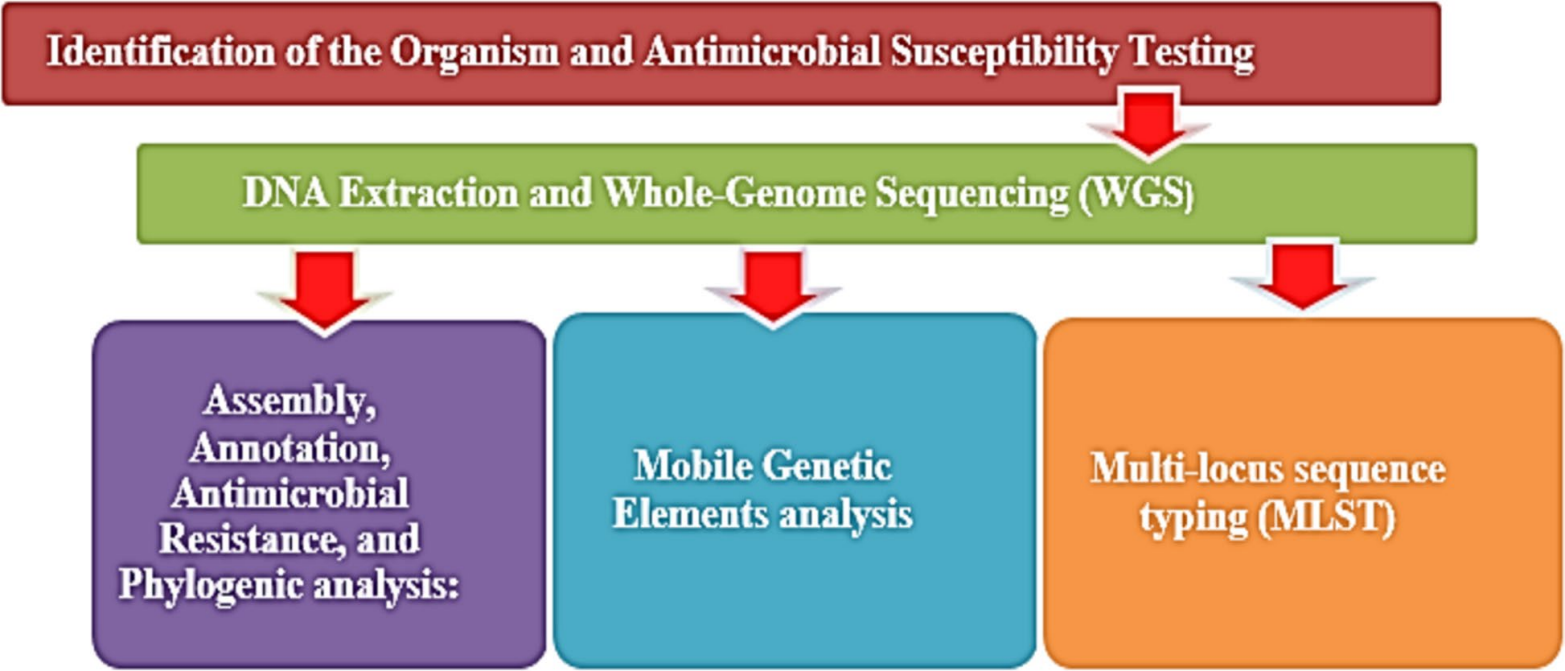
Workflow of the study

The current observational cross-sectional analytic study was performed on two *A. baumannii* strains. They were isolated from endotracheal aspirates (ETA) from two neonates admitted at the NICU, Ain Shams University Hospital at different times. Both patients presented with clinical signs of pneumonia that required NICU admission and died after short period of admission (Fig. 1).

### Identification of the organism

ETA collected from patients was sent immediately to the microbiology laboratory for processing. They were cultured on MacConkey agar medium (Oxoid, UK). Bacterial colonies were identified using conventional phenotypic identification including; culture morphology, Gram staining and biochemical reactions. VITEK II compact bacterial system (bioMerieux-Marcy-l’Étoile-France) was used to confirm isolates identification [23].

### Antimicrobial susceptibility testing

Antimicrobial susceptibility testing was performed using disc diffusion method (Kirby-Bauer) and minimal inhibitory concentration (MIC) following guidelines of Clinical and Laboratory Standards Institute (CLSI) guidelines [24].

#### Disc diffusion method (Kirby-Bauer)

Antibiotic discs (delivered from Oxoid, England) containing the subsequent drug concentrations were utilized: Ceftazidime (30 ug), Cefotaxime (30 ug), Cefepime (30 ug), Ceftriaxone (30 ug), Imipenem (10 ug), Aztreonam (30 ug), Piperacillin + Tazobactam (100/10 ug), Meropenem (10 ug), Ampicillin + Sulbactam (10 /10 ug), Gentamicin (10 ug), Tetracycline (30 ug), Amikacin (30 ug), Tigecycline (15 ug), Ciprofloxacin (5 ug), Levofloxacin (5 ug), Trimethoprim + Sulfamethoxazole (1.25/23.75 ug). The results were analyzed utilizing the CLSI breakpoints for all antibiotics (except for tigecycline) [24]. The results of tigecycline were analyzed following the Food and Drug Administration (FDA) breakpoints [25].

#### Minimal inhibitory concentration

The automated VITEK 2 compact system was utilized to detect MIC of all tested antibiotics, except colistin, following CLSI breakpoints [24]. The broth microdilution technique was used to determine MIC of colistin, following the European Committee on Antimicrobial Susceptibility Testing (EUCAST) breakpoints [26]. The following concentration range of antibiotics was used; Ampicillin + Sulbactam, Ceftazidime, Cefotaxime, Cefepime, Ceftriaxone, Aztreonam, Tetracycline, Amikacin, Ciprofloxacin, Levofloxacin, Gentamicin (0.5–256 ug/mL), Imipenem and Meropenem (0.06–32 ug/mL), Piperacillin + Tazobactam (0.5–512 ug/mL), Trimethoprim + Sulfamethoxazole (4–128 ug/mL), Tigecycline (0.125 -128 μg/mL), Colistin (0.25- 4 ug/mL).

### DNA Extraction and WGS

Two clinical *A. baumannii* isolates underwent WGS. One milliliter of an overnight bacterial culture was utilized for extracting the total genome including the chromosomal and extrachromosomal entities. MagMAX Microbiome Ultra Nucleic Acid Isolation kit (Applied Biosystems & ThermoFisher Scientific- Monza, Italy) was utilized following the instructions provided by the manufacturer. DNA concentrations have been determined using a Qubit fluorometer (ThermoFisher Scientific) to estimate DNA input.

WGS was performed using Illumina MiSeq (REF SY-410–1003) and Nextera XT-DNA library prep kit per the manufacturer's instructions. The library was sequenced at the Next Generation Sequencing Unit, Biological Prevention Department, Ministry of Defense, Egypt.

### Assembly, annotation, antimicrobial resistance, and phylogenic analysis

The two clinical *A. baumannii* reads were submitted to NCBI, and accession numbers were obtained (SRR26868873, SRR26868872). The comprehensive genome analysis was subsequently done using PATRIC [27] using the annotation statistics, followed by comparing it to other PATRIC genomes with *A. baumannii* (Tax ID:470). After that, genome annotations were done utilizing the RAST tool kit (RASTtk) [28].

ResFinder and the k-mer-based AMR genes detection method were utilized for the identification of AMR genes. This method uses PATRIC's curated collection of representative AMR gene sequence variants and provides functional annotations and broad antibiotic resistance mechanisms (for each AMR gene).

The phylogenetic analysis was conducted using the closest reference as well as representative genomes that were identified through the Mash/MinHash method [29]. This genome's phylogenetic placement was identified by choosing PATRIC global protein families (PGFams) [30]. These families' protein sequences were aligned using MUSCLE [31], and each sequence's nucleotides were matched to the protein alignment. Subsequently, nucleotide and amino acid alignments were combined to create a data matrix, which was analyzed utilizing RaxML [32]. Fast bootstrapping (100 bootstrap) was also utilized to obtain the support values (in the tree) [33].

### MGEs analysis

The identification of AMR genes associated with MGEs was carried out using a CGE server [34], which accurately predicts the mobility and rapid dissemination of these elements within a bacterial community. Mobile Element Finder was developed to rapidly detect MGEs (in addition to their genetic context) in assembled sequence data.

### Multilocus sequence typing (MLST)

MLST on genomes of collected *A. baumannii* was done using the Oxoford scheme. This scheme entails identifying seven internal housekeeping genes: RNA polymerase _70factor (*rpoD*), glucose-6-phosphate isomerase (*gpi*), glucose dehydrogenase B (*gdhB*), DNA gyrase subunit B (*gyrB*), citrate synthase (*gltA*), 60-kDa chaperonin(*cpn60*), and homologous recombination factor (*recA)* [35].

## Results

Two clinical *A. baumannii* strains (Ab119 and Ab123) were isolated from ETA from two neonates presented with pneumonia and died at NICU at Ain Shams University Hospital.

### Antimicrobial susceptibility testing

Both isolates exhibited resistance to all used antibiotics (except for tigecycline & colistin). MIC results showed a remarkable resistance to trimethoprim–sulfamethoxazole, gentamicin, and ciprofloxacin (Table 1).

**Table 1:** MICs of antibiotics used for Ab 119 and Ab 123 isolates

Antibiotics	MIC ug/mL	
	Ab119	Ab 123
**Ceftazidime**	32	32
**Cefotaxime**	128	64
**Ceftriaxone**	64	64
**Cefepime**	64	32
**Imipenem**	8	16
**Meropenem**	8	8
**Piperacillin + Tazobactam**	128	256
**Ampicillin + Sulbactam**	32	64
**Gentamicin**	32	64
**Amikacin**	32	64
**Tetracycline**	16	32
**Ciprofloxacin**	128	64
**Levofloxacin**	16	32
**Trimethoprim + Sulfamethoxazole**	4	8
**Tigecycline**	2	1
**Colistin**	1	1

### WGS and MLST

**Fig. 2:**
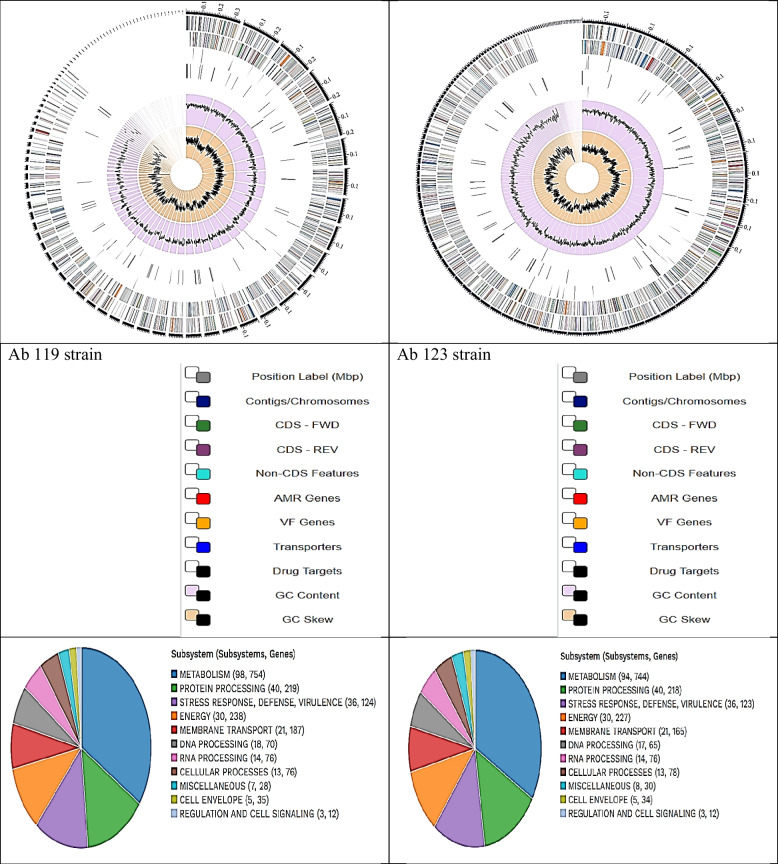
Circular graphical display of the genome annotation distribution. The figure includes, from outer to inner rings, the contigs, CDS on the forward strand, CDS on the reverse strand, RNA genes, CDS with homology to known antimicrobial resistance genes, CDS with homology to know virulence factors, GC content and GC skew. The colors of the CDS on the forward and reverse strand indicate the subsystem that these genes belong to

According to the annotation statistics and comparing the genome of *A. baumannii* (Tax ID: 470) in PATRIC. The genomes exhibited excellent quality. The bioinformatics and data analysis of both strains are shown in Table 2 and Fig. 2.

**Table 2:** Genome characteristics of Ab 119 and Ab 123 strains

Strain	Contigs	Genome Length (bp)	GC Content %))	Protein CDS	transfer RNA	ribosomal RNA
Ab119	92	4,251,654	38.80	4,226	63	3
Ab123	132	4,097,941	38.84	4,017	63	3

The MLST analysis utilizing the Oxford scheme indicated that both isolates belong to the ST931which is related to IC3.

### Phylogenetic analysis

**Fig. 3:**
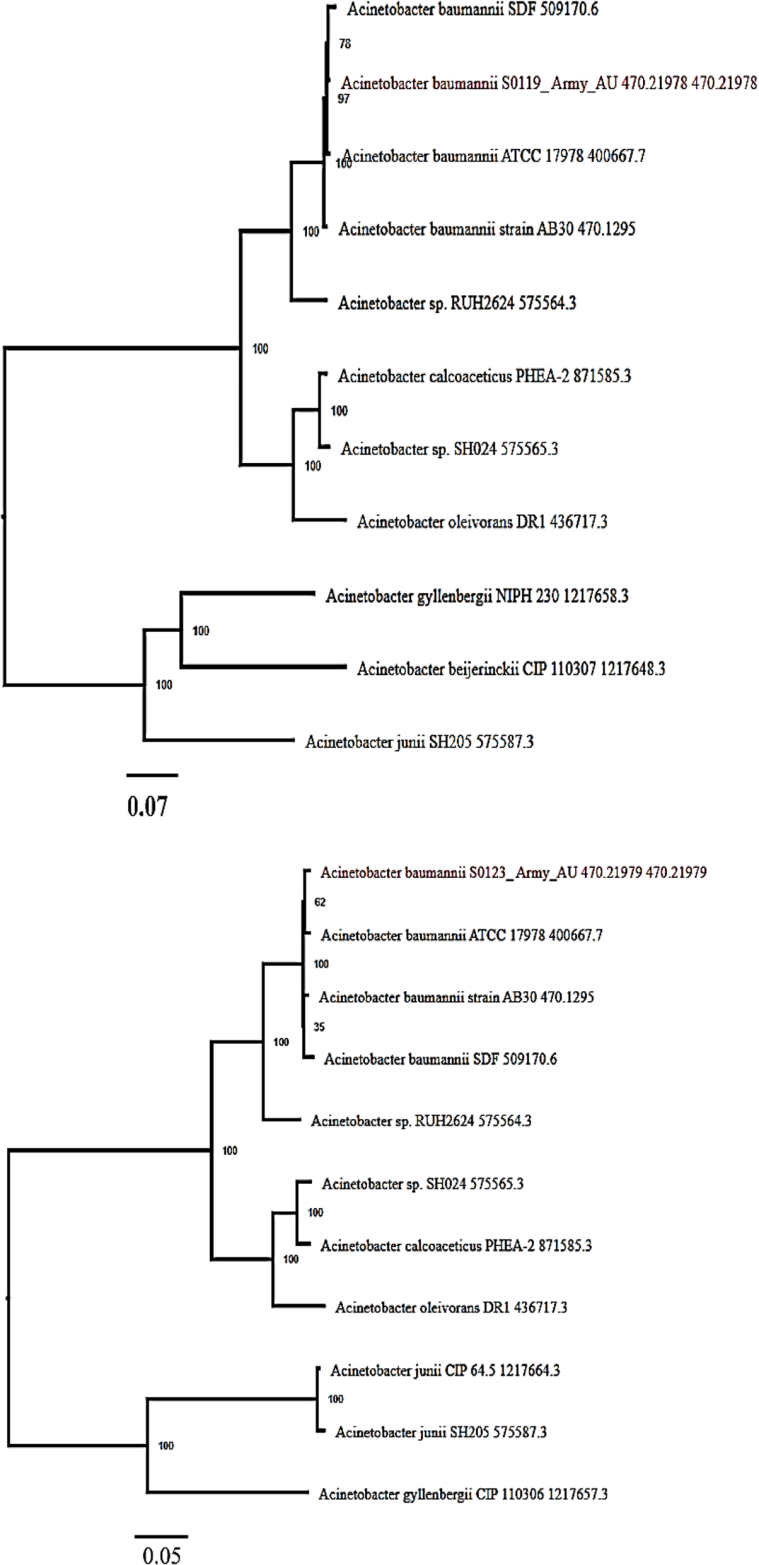
Phylogenetic tree of Ab 119 and Ab 123 strains. Phylogenetic analysis was done with closest reference and representative genomes identified by Mash/MinHash. PATRIC global protein families, PGFams were selected from these genomes to determine the phylogenetic placement of this genome. The protein sequences from these families were aligned with MUSCLE and the nucleotides for each of those sequences were mapped to the protein alignment. The joint set of amino acid and nucleotide alignments was concatenated into a data matrix, and RaxML was used to analyze this matrix, with 100 bootstrapping was used to generate the support values in the tree

The phylogenetic analysis of the samples showed a close similarity to *A. baumannii* SDF 509170.6 and *A. baumannii* ATCC 17978 400,667.7, with the Ab119 and Ab123 strains, respectively (Fig. 3).

### Antimicrobial Resistance Genes (AMR)

Predicted antimicrobial resistance phenotypes analysis showed multidrug resistance for both isolates. ResFinder analysis revealed different genes as:

- β-Lactam resistance genes: both isolates harbored molecular class D and class C β-lactamases. In general, *bla OXA-23* and *bla ADC-25* genes were detected in both isolates with a copresence of *bla OXA-64, bla NDM-10,* and *bla NDM-1* in the AB 119 strain. The presence of bla OXA-68, bla PER-7, and bla PER-1 was detected in the AB 123 strain.
- Aminoglycoside resistance genes: The strains exhibited resistance to aminoglycosides because of the detection of the following genes: armA, aph(3')-Via, ant(2'')-Ia, aph(3'')-Ib, and aph(6)-Id. The AB 119 strain exhibited the simultaneous aph(3')-VI gene presence.
- Macrolide resistance genes: Both isolates revealed *msr E* and *mph E* genes.
- Tetracycline resistance genes: Tetracycline resistance is mediated by tet B in the Ab119 strain and tet 39 in the Ab 123 strain.
- Other AMR genes: ARP 2 and ARP 3 (rifamycin resistance), Sul 2 and sul1 (sulfonamide resistance), in addition to cmlA1 (chloramphenicol resistance) genes, were found in both isolates (Table 3).

**Table 3:** AMR genes present in Ab 119 and Ab 123 strains

Strain	B-Lactam resistance genes	Aminoglycoside resistance genes	Macrolide resistance genes	Tetracycline resistance genes	Others: *Rifamycin *Sulfamethoxazole * Chloramphenicol
Ab 119	*blaNDM-1* *blaNDM-10* *blaADC-25* *blaOXA-23* *blaOXA-64*	*aph(3')-VI* *arm A* *aph(3')-*Via *ant(2'')-Ia* *aph(3'')-Ib* *aph(6)-Id*	*Msr E* *Mph E*	*Tet B*	*ARR2* *ARR 3* *sul2* *sul1* *cmlA1*
Ab123	*blaOXA-68* *blaADC-25* *blaOXA-23* *blaPER-7* *blaPER-1*	*ant(2'')-Ia* *aph(3'')-Ib* *aph(6)-Id* *armA* *aph(3')-*Via	*Msr E* *Mph E*	*Tet 39*	*ARR 2* *ARR 3* *sul2* *sul1* *cmlA1*

The CARD platform was utilized to conduct k-mer prediction of the pathogen responsible for AMR genes. This online platform facilitates the examination of metagenomic contigs, genome assemblies, and genomes. CARD’s RGI (Resistance Gene Identifier) allows predictions resistomes from protein or nucleotide data based on homology model with defined criteria ranging from perfect and strict matches to loose similarities [36].

**Fig. 4:**
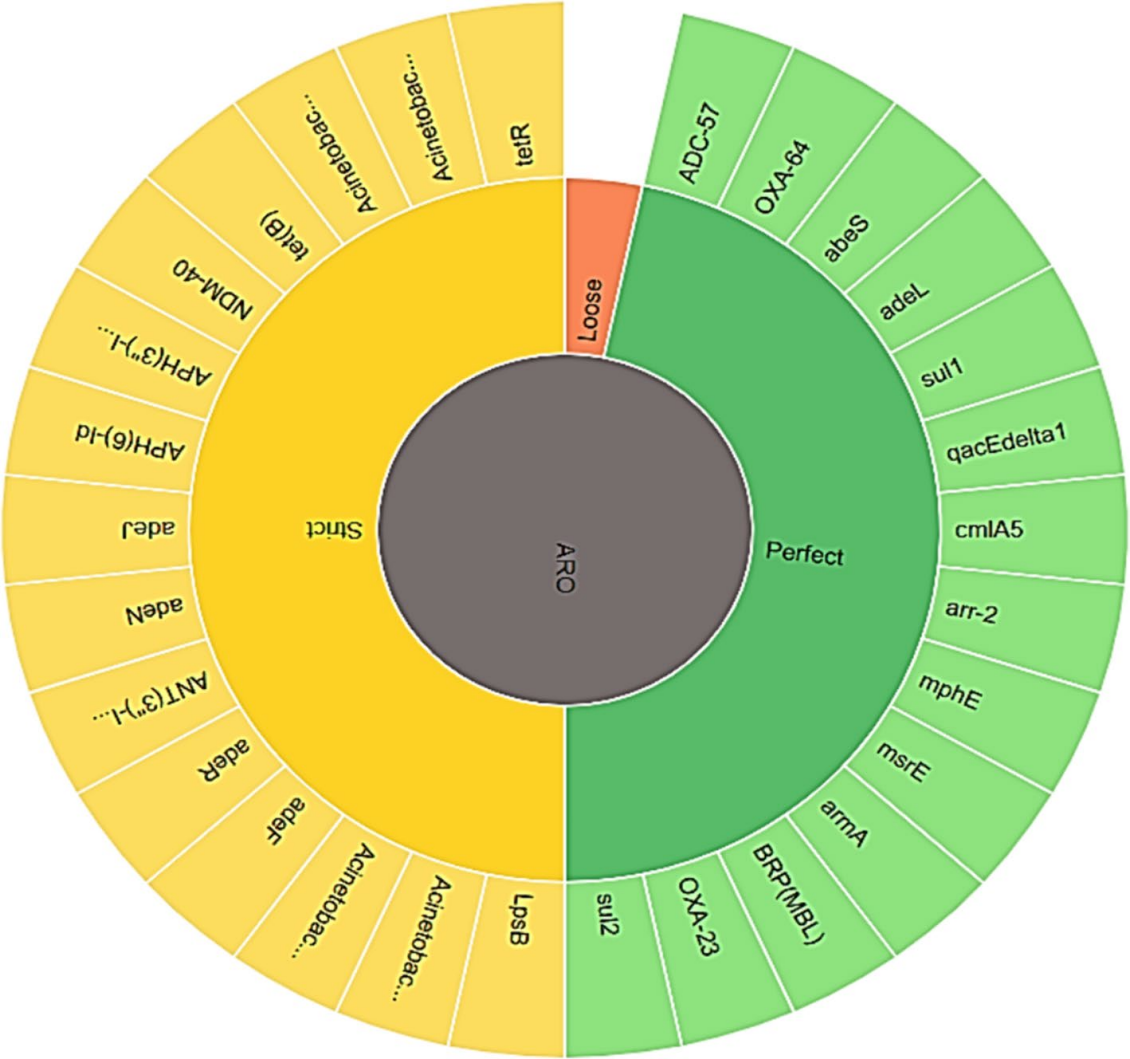
Antimicrobial resistance gene origin in Ab119 strain. The figure illustrates the classification of antimicrobial resistance gene origin including three types according to their ordering in genetic identity from high to low (14 genes predicted to be perfect, 15 genes to be strict and none to be loose)

Regarding the Ab119 strain, origin of AMR genes, resistance mechanisms, and drugs were revealed, as shown in (Fig. 4, Table 4). The protein homolog model was found in all genes except for *gyr A* (S81L), and *ParC* (V104I and D105E) mutations were detected, as well as the *tet* R gene overexpression.

**Table 4:** AMR genes' origin, drug affected, and resistance mechanisms shown in the Ab 119 strain

*RGI Criteria	**ARO Term	*Drug class affected*	*Resistance mechanism*
***Perfect	*ADC-57*	*Cephalosporin*	*Antibiotic-inactivation*
	*OXA-64*	*carbapenem, cephalosporin*	*Antibiotic-inactivation*
	*abeS*	*macrolide, aminocoumarin*	*Antibiotic*-efflux
	*adeL*	*fluoroquinolone, tetracycline*	*Antibiotic*-efflux
	*sul1*	*Sulfonamide*	Antibiotic-target *replacement*
	*qacEdelta1*	antiseptics & disinfecting agents	*Antibiotic*-efflux
	*cmlA5*	*Phenicol*	*Antibiotic*-efflux
	*arr-2*	*Rifamycin*	*Antibiotic-inactivation*
	*mphE*	*Macrolide*	Antibiotic-inactivation
	*msrE*	macrolide, streptogramin	*Antibiotic*-target protection
	*armA*	*Aminoglycoside*	*Antibiotic-target alteration*
	*BRP(MBL)*	*Glycopeptide*	Antibiotic-inactivation
	*OXA-23*	*carbapenem, cephalosporin*	*Antibiotic-inactivation*
	*sul2*	*Sulfonamide*	*Antibiotic*-target replacement
***Strict	*LpsB*	*Peptide*	*Reduced-permeability to antibiotics*
	*A. baumannii AmvA*	*macrolide, disinfecting agents and antiseptics*	Antibiotic-efflux
	*A. baumannii AbaF*	*phosphonic acid*	*Antibiotic*-efflux
	*adeF*	*fluoroquinolone, tetracycline*	*Antibiotic*-efflux
	*adeR*	*glycylcycline, tetracycline*	Antibiotic-efflux
	*ANT(3'')-IIc*	*Aminoglycoside*	Antibiotic-inactivation
	*adeN*	Macrolide- diaminopyrimidine lincosamide- *fluoroquinolone*-carbapenem- tetracycline-cephalosporin- rifamycin-phenicol	Antibiotic-efflux
	*adeJ*	Macrolide- lincosamide- fluoroquinolone-*carbapenem*-cephalosporin- rifamycin- tetracycline- phenicol- diaminopyrimidine-	Antibiotic-efflux
	*APH(6)-Id*	*Aminoglycoside*	*Antibiotic-inactivation*
	*APH(3'')-Ib*	*Aminoglycoside*	*Antibiotic-inactivation*
	*NDM-40*	carbapenem, cephalosporin, cephamycin	Antibiotic-inactivation
	*tet(B)*	*Tetracycline*	*Antibiotic*-efflux
	*A. baumannii gyrA*	*Fluoroquinolone*	*Antibiotic-target alteration*
	*A. baumannii parC*	*Fluoroquinolone*	Antibiotic-target *alteration*
***Strict	*tetR*	*Tetracycline*	*Antibiotic-target alteration* *Antibiotic-efflux*

^*^RGI Resistance Gene Identifier

^**^ARO Antibiotic Resistance Ontology

^***^Perfect and strict (i.e.; with high confidence and probability)

**Fig. 5:**
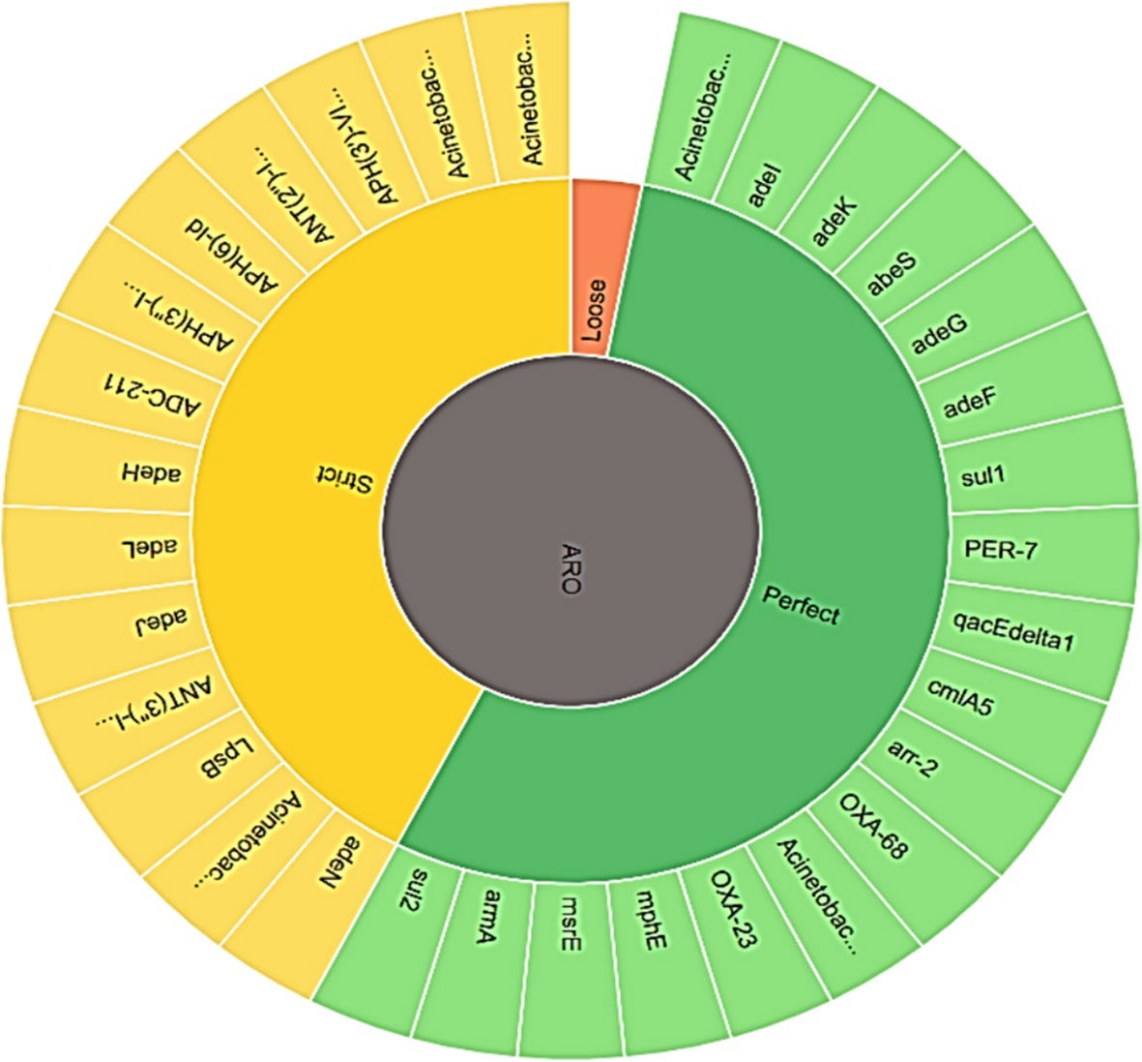
Antimicrobial resistance gene origin in Ab 123 strain. The figure illustrates the classification of antimicrobial resistance gene origin including three types according to their ordering in genetic identity from high to low (18 genes predicted to be perfect, 14 genes to be strict and none to be loose)

Regarding the Ab123 strain, the origin of AMR genes, resistance mechanisms, and drugs were revealed and shown in (Fig. 5, Table 5). The protein homolog model was found in all genes except for *gyr A* (S81L), and *ParC* (S84L, V104I, and D105E) mutations were detected.

**Table 5:** AMR genes origin, drug affected, and resistance mechanisms shown in Ab123 strain

***RGI*** Criteria	ARO term	***Drug class***	Resistance mechanisms
Perfect	*A.* baumannii *AbaF*	phosphonic acid	Antibiotic-efflux
	*adeI* *adeK*	Macrolide-fluoroquinolone-lincosamide- carbapenem- tetracycline- cephalosporin-rifamycin-phenicol- diaminopyrimidine	Antibiotic-efflux
	*abeS*	macrolide, aminocoumarin	Antibiotic-efflux
	*adeG* *adeF*	Fluoroquinolone- tetracycline	Antibiotic-efflux
	*sul1*	sulfonamide	Antibiotic-target replacement
	PER*-7*	Monobactam-carbapenem- cephalosporin	Antibiotic-inactivation
	*sul1*	sulfonamide	Antibiotic-target replacement
	*qacEdelta1*	disinfecting agents and antiseptics	Antibiotic-efflux
	cmlA5	phenicol	Antibiotic-efflux
	arr-2	rifamycin	Antibiotic-inactivation
	*OXA-68*	carbapenem, cephalosporin	Antibiotic-inactivation
	A. baumannii AbaQ	fluoroquinolone	Antibiotic-efflux
	*OXA-23*	carbapenem, cephalosporin	Antibiotic-inactivation
	mphE	macrolide	Antibiotic-inactivation
	msrE	macrolide-streptogramin	Antibiotic-target protection
	armA	aminoglycoside	Antibiotic-target alteration
	sul2	sulfonamide	Antibiotic-target replacement
**Strict**	adeN adeJ	Macrolide- lincosamide- fluoroquinolone- cephalosporin-carbapenem- tetracycline- diaminopyrimidine- rifamycin-phenicol	Antibiotic-efflux
	*A.* baumannii *AmvA*	Antiseptics- disinfecting agents- macrolide	Antibiotic-efflux
	LpsB	peptide	Reduced permeability to antibiotics
	*ANT(3'')-IIc*	aminoglycoside	Antibiotic-inactivation
	*adeL* *adeH*	fluoroquinolone, tetracycline	Antibiotic-efflux
	*ADC-*211	Cephalosporin	Antibiotic-inactivation
	*APH(*3''*)-Ib*	aminoglycoside antibiotic	Antibiotic-inactivation
	APH*(6)-Id*	aminoglycoside	Antibiotic-inactivation
	*ANT(*2''*)-Ia*	aminoglycoside	Antibiotic-inactivation
	*APH(3')-*Via	aminoglycoside	Antibiotic-inactivation
	*A.* baumannii *gyrA*	fluoroquinolone	Antibiotic-target alteration
	*A. baumannii* parC	fluoroquinolone	Antibiotic-target alteration

### Mobile Genetic Elements (MGEs)

MGEs were identified by comparing their sequences to a database of (4,452) known elements. This database was enhanced with information about virulence factors, resistance genes, and the identification of plasmids. The analysis of strains indicated the presence of a diverse range of MGE, insertion sequences (ISs) that belong to different IS families. In the Ab119 strain, 14 MGE were detected. Conversely, only 10 MGE were detected in the Ab123 strain. Both strains showed the presence of (IS*Ec29* related to *armA* and *msrE),* (IS*Ec28* related to *sul1*, *Arr-2* and *bla PER-7*) and (IS1007, CN-10921-IS1007 related to *sul1*). In addition, Ab123 strain showed (IS*Vsa3* that related to *aph(3)-Ib* and *aph(6)-Id*). Other ISs that aren't linked to ARG were detected in both strains as (IS*Aba14*, IS*Aba34*, IS*Aba37*, IS1008, IS*Aca1*). Others were detected only in Ab119 strain as (IS *Aba10*, IS*Aba33*, IS26, IS*Vsa3*). No plasmids were detected in both isolates as shown in Tables 6 and 7.

**Table 6:**
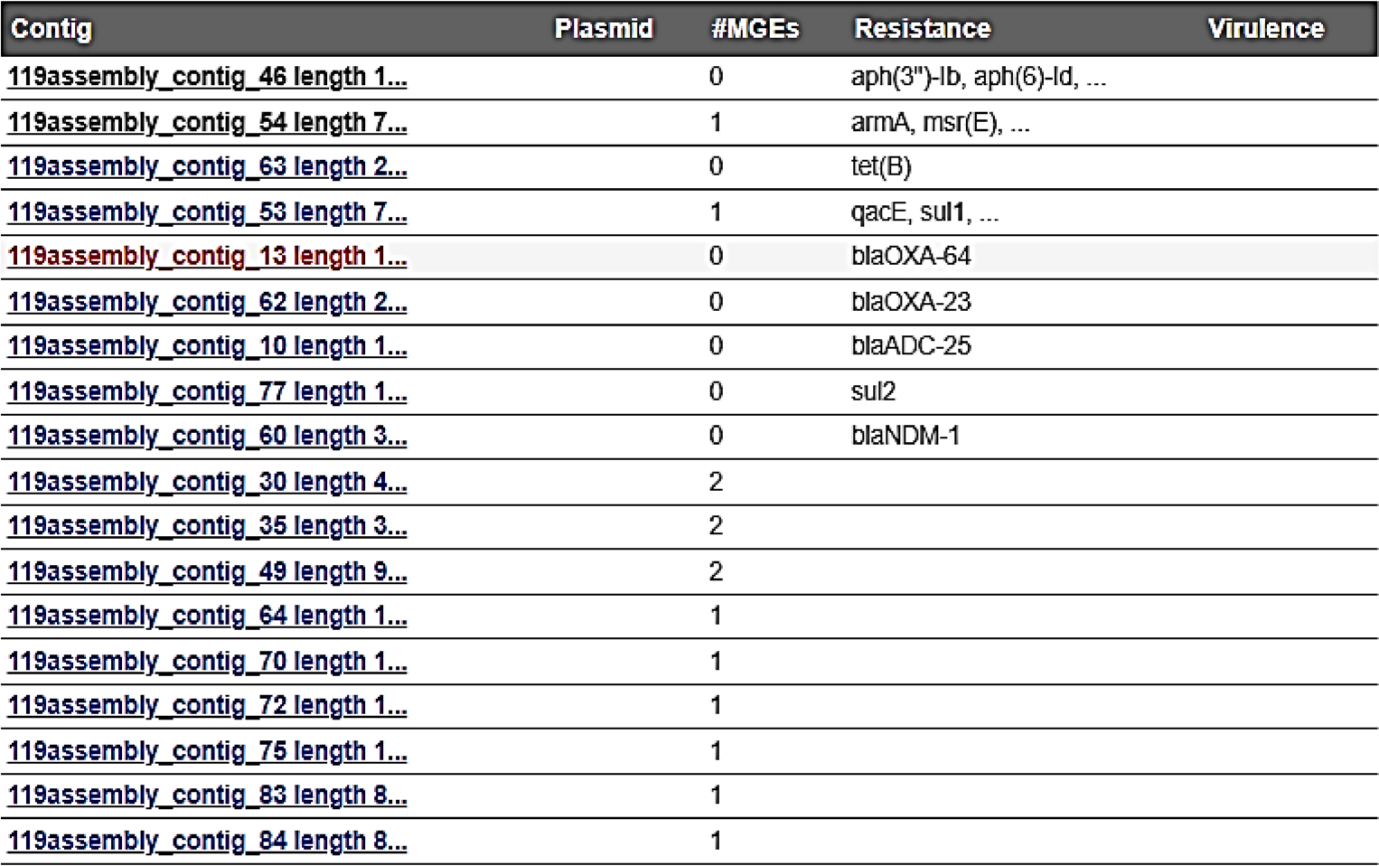
Mobile genetic elements (14 MGE) in Ab 119 strain

**Table 7:**
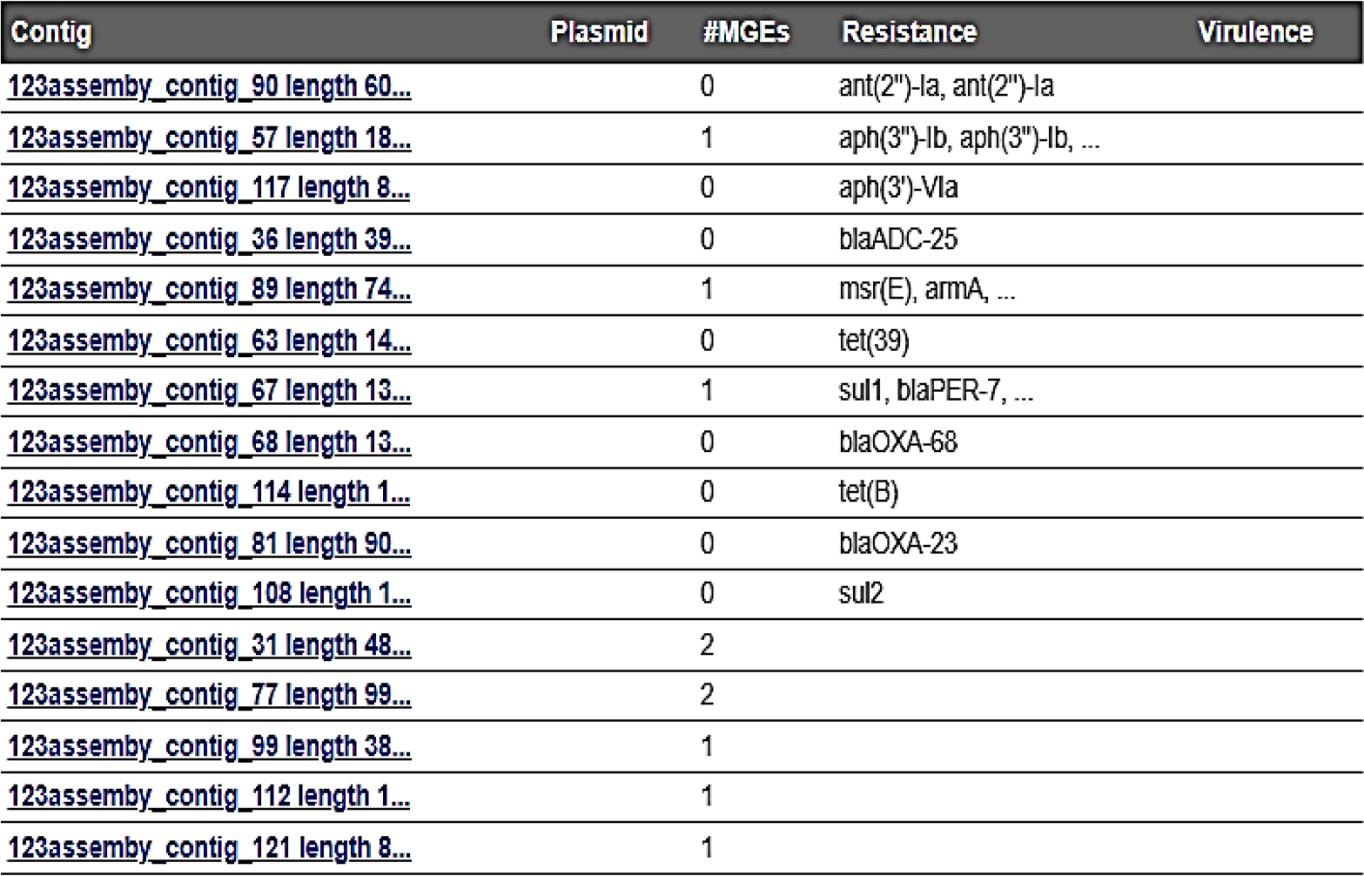
Mobile genetic elements (10 MGE) in Ab123 strain

## Discussion

*A. baumannii* can persist in different environmental circumstances and develop AMR, making it one of hospitals' most life-threatening nosocomial pathogens [37]. MDR nosocomial *A. baumannii* can cause severe infections in neonates with an increased number of mortalities. The presence of foreign DNA [21, 22] and different plasmids in its genome increase the gaining of AMR genes [20]. Investigating AMR genes among *A. baumannii* clinical isolates is mandatory for revealing the incriminated mechanisms of AMR development. Specific Egyptian laboratories have access to new methods like NGS technology, which can replace conventional PCR. These methods are used to identify AMR genes. This study focuses on identifying AMR profiles and AMR genes of two clinical isolates of *A. baumannii*. These isolates were obtained from ETA samples of two neonates admitted to the NICU at ASU Hospital, a large tertiary care hospital in Egypt.

The current investigation demonstrated that both strains exhibited sensitivity to tigecycline and colistin while displaying resistance to all other antibiotics that were tested, encompassing third-generation cephalosporin, carbapenems, aminoglycosides, fluoroquinolones, and trimethoprim/ sulfamethoxazole. The obtained outcomes closely align with the findings of Gaafar et al. (2022), who conducted a study on neonates with sepsis at Zagazig University Hospitals. They reported that *Acinetobacter* isolates were most sensitive to ciprofloxacin, colistin, and tigecycline, while they were most resistant to trimethoprim/ sulfamethoxazole [38]. In India, a study conducted by Nazir in 2019 found that 95.9% of Acinetobacter strains isolated from the NICU showed resistance to aminoglycosides, cephalosporins, penicillin, and fluoroquinolones. Additionally, 93.68% of the strains exhibited resistance to carbapenems [39].

Carbapenems, even in neonates, are considered first-line therapy for lower respiratory tract infections. However, increased resistance to nearly all existing antimicrobial agents including carbapenems is alarming.

Although our study revealed sensitivity to colistin and tigecycline, studies issued in 2020 reported that about 53% of* A. baumannii* Egyptian isolates were colistin-resistant [40, 41]. In addition, tigecycline nonsusceptibility was reported in many studies in Egypt [42, 43]. Differences in resistance pattern may be due to restricted usages of colistin and tigecycline at NICU. Moreover, the low isolate number incorporated in our study may cause this difference.

Sequenced-genome analysis indicated that both isolates belonged to the same MLST sequence type (ST931), a representative of the (ST52) pasture scheme, and belongs to international clone 3 (IC3) [44]. A limited number of reports have documented the occurrence of this type globally [45]. It was incriminated in the Netherlands outbreak in 1986 [46]. In 2015, another study documented the occurrence of this type in Nepal [47].

To our knowledge, only one previous study in Egypt reported ST931 in a single isolate among 45* A. baumannii* isolates in Tanta University Hospital in 2015. The authors declared that most isolates belonged to IC2 and IC1 [48]. Different studies in various places in the Middle East and Egypt [49–52] reported the spread of IC2. Furthermore, IC1&IC2 were found in all continents, indicating their global distribution, and they frequently contain the acquired carbapenemase genes [53–55].

Nevertheless, IC3 was previously regarded as a prominent clone. However, its occurrence has significantly decreased in the past ten years, and there have been very few documented cases of it originating from non-human sources [56, 57]. IC3 has had limited significance in recent years, with occasional instances reported in Peru [58], South Africa, the USA, and Spain [44].

Our finding that both isolates belonged to the same sequence type (ST931) indicates that this sequence type which belongs to the IC3 group, is likely to start spreading in Egypt and has the potential to become widespread. It is crucial to consider the introduction of a low prevalent sequence type that has a high rate of resistance and transmission into healthcare settings.

A total of 32 and 33 antibiotic resistance genes (ARG) were detected in the Ab119 and Ab123 strains, respectively, through genome sequencing, ResFinder, and CARD platform analysis. Distinct genetic factors that confer resistance to cephalosporins and carbapenems, class A, C, and D β-lactamases, were identified. Neither strain exhibited the presence of KPC nor any other MBLs such as IMP, SPM, VIM, or SIM. Additionally, no other OXA families were detected, including OXA-58. Reporting that each strain carries genes of three classes of beta-lactamase is highly unfavorable to clinicians due to high failure rates of treatment with beta-lactams.

Our results are comparable to those of various studies conducted in tertiary hospitals in Egypt. A study was conducted on carbapenem-resistant *A. baumannii* (CR-AB) isolated from multiple clinical units at Kasr Al-Aini Hospital. They reported that the most predominant beta-lactamase gene type among isolates was *blaOXA-23*, followed by *blaNDM-1* and *blaKPC*. They reported low prevalence of other genes such as *bla SPM* 6.3%, *OXA -58* 1.9%, *bla VIM* 0.5%, and *bla SIM* 0.5%. The low number of isolates, 18.4%, harbored two or more *bla* genes [59].

Another study was conducted on carbapenem-insensitive *A. baumannii* strains isolated from two hospitals, Dar el-Foad and Kasr Al-Aini, Egypt. The *bla ADC and blaOXA-51*-like genes were observed in all isolates. The prevalence of *bla OXA-23, blaPER* was 50% and 55%, respectively. However, no isolate carried KPC or MBL-encoding genes [60].

Moreover, a prior study (Zagazig University) stated that 90% and 66.7% of CRAB isolates carried *blaOXA-23* and NDM, respectively [61]. Other studies in KSA and Egypt reported that all CRAB isolates harbored *blaOXA-23* [62, 63]. They reported different rates 12.1% and 100% of *blaNDM* and *blaVIM* [62]. Previous studies in Egypt revealed two variants of NDM (NDM-1 & NDM-2) among *A. baumannii* clinical isolates [64, 65].

These results denoted a significant prevalence of OXA 23 and NDM in various hospitals in Egypt, particularly in tertiary hospitals. This evidence confirms that class D β-lactamases are the prevailing type of carbapenemases, with MBLs being the second most prevalent. To the best of our knowledge, this is the initial instance of documenting the presence of NDM-10 and NDM-40 in Egyptian hospitals. NDM-positive strains are correlated with severe consequences as well as increased mortalities, especially in neonates and immunosuppressed individuals. Various infections caused by these strains have been documented to have unfavorable outcomes [66], which is alarming for the spread of NDM-harboring isolates in the NICU.

The discrepancies observed in different studies can be attributable to the fact that some authors did not examine all genomes and instead employed multiplex PCR to probe for the detection of particular genes specifically.

The present study uncovered several genes that provide resistance to aminoglycoside in both strains. The *armA* gene confers resistance to gentamicin, while *ant(2'')-Ia* mediates resistance to gentamycin, kanamycin, and tobramycin. *Aph (3')-*VIa* and aph(3')-VI* seem to confer resistance to amikacin and kanamycin.

Comparable results were reported by ELsheredy et al., who illustrated that *A. baumannii* clinical isolates from cases in multiple ICUs in Alexandria University Hospital harbored *armA* and AME genes (aphA*6, aphA1*). However, they reported other AME genes as *aacA4, aacC1, aadA1, and aadB* [67]. Another study reported that most isolates carried genes conferring resistance to aminoglycosides (*strA, aadA1-pm, armA, strB, aph(3')-VI, aph(3')-Ia, aph(3')-*VIa*, ant(3")-II, aac(6')-Ib, and ant(3")-IIa*) [68].

The combination of these multiple genes limits the usage of nearly all aminoglycosides as an alternative therapy for *A. baumannii* and makes treatment highly challenging.

In addition, our study revealed that both isolates harbored resistance genes to tetracyclines. *Tet B* is a tetracycline efflux protein found in various Gram-negative bacteria. The major facilitator superfamily (MFS) antibiotic efflux pump provides resistance to tetracycline, minocycline, and doxycycline (but not tigecycline) [69]. *Tet39* confers tetracycline and doxycycline resistance only [70].

*Sul1* and *Sul2* genes were revealed in both isolates and mediated resistance to sulfonamides. Transposons or plasmids in most Gram-negative bacteria carry these genes, which provide resistance to sulfonamides. Isolates can develop resistance to trimethoprim/sulfamethoxazole through one or both genes [71].

Both isolates in our study carried the *cmlA1* gene, which encodes chloramphenicol acetyltransferase and confers resistance to chloramphenicol. Earlier reports indicated that the majority of *A. baumannii* isolates have inherent resistance to chloramphenicol. However, the mechanisms responsible for this resistance have not been evident until now [72].

Furthermore, both isolates exhibited the presence of *mphE* and *msrE* genes, which confer resistance to macrolides, as well as the *arr2* gene, which confers resistance to rifamycin. Similar genes were detected by Sa' nchez-Urtaza et al. in Alex; *tet39*, *tetB, sul1, msr.E, sul2*, *cmlA5, mph.E*, and *arr-2*. However, they reported the presence of other genes, such as *catB8* and *catA1* that mediate resistance to chloramphenicol and *dfrA7* that confer resistance to trimethoprim [68].

Efflux pumps are incriminated into increased antibiotic resistance in *A. baumannii*. In our study, we revealed different efflux pump coding genesbelonging to the SMR family, the RND family, and the MFS family. Based on our understanding, these genes encode efflux pumps that play a crucial role in increasing resistance to various antibiotics, particularly fluoroquinolones and tetracyclines.

Comparable results were reported by Sánchez-Urtaza et al. in Alex. They declared that most isolates harbored efflux pump encoding genes, as detected in our results, in addition to *abeM, adeA, adeB, adeC, and adeS* [68]*.*

Both sequenced isolates also detected mutation in *gyrA* (S81L) and *par C* (V104I, D105E and S84L). Kumburu et al. reported similar *gyr A* and *par C* (S84L) mutations in 50% of the studied isolates. Segatore et al. reported similar mutations in *gyrA* and *parC* (V104I, D105E) in *A. baumannii* isolated from a multicenter in Italy [73].

Different studies from various hospitals in Egypt detected other mutations in *gyrA* (S83L) and *parC* (S80L) among fluoroquinolone-resistant *A. baumannii* isolates. Their prevalence ranged from 100%, as illustrated by Tantawy et al. [74] and Zaki et al. [75], to 23.7%, as reported by Taha et al. [76].

However, the primary mechanism of fluoroquinolone resistance typically involves mutations in the quinolone resistance determining region of (*gyrA*) that encodes the DNA gyrase A and (*parC*) that encodes the topoisomerase IV. The isolates with triple and quadruple mutations exhibited a significantly elevated resistance to ciprofloxacin and levofloxacin. To our knowledge, it is the first time these mutations in *A. baumannii* (isolated from Egyptian hospitals) have been detected.

We observed that specific genes were not identified when using the ResFinder server but were identified when utilizing the CARD databases. Therefore, our findings have confirmed the necessity of utilizing multiple databases to ascertain the resistance profiles of bacterial isolates to prevent the missing of specific resistance genes.

Both isolates show the presence of multiple ISs associated to *armA, msrE, and sul1* genes. In addition, the Ab123 isolate harbored ISs associated to *aph(3)-Ib* and *blaPER7* genes. Numerous MGEs have been discovered in different bacteria, including *A. baumannii.* Nevertheless, the existence of these mobile elements confers a significant degree of resistance to various antibiotics and is implicated in the process of horizontal gene transfer within bacterial cells and between different cells. Furthermore, it is accountable for acquiring new properties, such as antibiotic resistance and pathogenicity [77].

Although various genes such as *NDM,* some *OXA*, *armA, ant(2'')-Ia, aph(3')-*Via*, aph(3'')-Ib, aph(6)-Id* are known to be plasmid-mediated [78], our results detected these genes on bacterial chromosome not on plasmid. This may be explained by MGEs as ISs and transposon found in plasmids can move and integrate into chromosome of the same or other bacterial cells causing spreading of resistance genes to new bacterial cells. MGEs are crucial in carrying and disseminating resistance genes, with a role comparable to that of plasmids.

## Conclusion

We identified a low-frequency strain of *A. baumannii* clone ST (931) in our clinical environment. This strain carries multiple resistance genes against all antibiotics, with the exception of colistin and tigecycline. The resistant isolates were not linked in terms of their epidemiology. New mutations were revealed *gyr A* (S81L) and (S84L, V104I and D105E) that did not report yet in Egypt but only in Europe. Isolates harbored multiple MGEs, but no plasmid was detected.

### Recommendation

Clinicians and healthcare workers must be aware of *A. baumannii* populations to implement suitable treatment and infection control protocols. Furthermore, the wide implementation of molecular and genomic technologies is crucial in order to obtain a precise epidemiological picture of *A. baumannii* and distinguish between different isolates, especially in tertiary health centers and ICUs that treat high-risk patients.

## Supplementary Information

Supplementary Material 1.

Supplementary Material 2.

## Data Availability

The datasets used and/or analyzed during the current study are available from the corresponding author on reasonable request. Data for both isolates were submitted on NCBI as SRA and accession numbers were obtained for ab119 isolate was SRR26868873 and ab 123 isolate was SRR26868872.

## Abbreviations

WGS: Whole-genome sequence
AMR: Antimicrobial resistance
ST: Sequence type
IC: International clone
MGEs: Mobile genetic elements
NICU: Neonatal intensive care unit
A. baumannii: Acinetobacter baumannii
ICUs: Intensive care units
ASU: Ain Shams University
PATRIC: Pathosystems Resources Integration Center
ETA: Endotracheal aspirates
MIC: Minimal inhibitory concentration
EUCAST: European Committee on Antimicrobial Susceptibility Testing
PGFams: Global protein families
MLST: Multilocus sequence typing
rpoD: RNA polymerase _70factor
Gpi: Glucose-6-phosphate isomerase
gdhB: Glucose dehydrogenase B
gyrB: DNA gyrase subunit B
gltA: Citrate synthase
recA: Homologous recombination factor
CDS: Protein-coding sequences
rRNA: Ribosomal RNA
RGI: Resistance Gene Identifier
ARO: Antibiotic Resistance Ontology
IS: Insertion Sequence
