# Supplementary material for: Whole-genome sequencing of two multidrug-resistant acinetobacter baumannii strains isolated from a neonatal intensive care unit in Egypt: a prospective cross-sectional study
Source: BMC Microbiol. 2024 Sep 21;24:362. doi: 10.1186/s12866-024-03482-3 (PMC11415996; doi:10.1186/s12866-024-03482-3)
Supplement: Supplementary file 1 — Supplementary Material 1. [file 12866_2024_3482_MOESM1_ESM.pdf]

4930662.cgebase.food.dtu.dk 4930663.cgebase.food.dtu.dk 4930664.cgebase.food.dtu.dk 4930665.cgebase.food.dtu.dk 4930666.cgebase.food.dtu.dk

Contig: 119assembly_contig_46 length 16949 coverage 123.1 normalized_cov 0.90

[illegible]

Gene name	Phenotype	Accession	Position in contig	Coverage	Identity
aph(3")-Ib	streptomycin	AF024602	1743-941	99.87562189054727%	100%
aph(6)-Id	streptomycin	M28829	105-941	100%	100%
aph(3")-Ib	streptomycin	AF313472	1744-941	100%	99.87562189054727%
aph(3")-Ib	streptomycin	AF321551	1744-941	100%	99.87562189054727%
aph(3")-Ib	streptomycin	AF321550	1744-941	100%	99.87562189054727%

Contig: 119assembly_contig_54 length 7438 coverage 147.0 normalized_cov 1.07

Resistance results

Gene name	Phenotype	Accession	Position in contig	Coverage	Identity
armA	amikacin, netilmicin, tobramycin, gentamicin, isepamicin	AY220558	6437-7210	100%	100%
msr(E)	quinupristin, virginiamycin s, pristinamycin ia, azithromycin, erythromycin	FR751518	2663-4138	100%	100%
mph(E)	erythromycin	DQ839391	1723-2607	100%	100%

ISEc29

Family	IS4
Group	IS10
Type	Insertion sequence
Reference db	isfinder
Accession	FJ187822
Position in contig	4503-5827
Strand	reverse
Alignment coverage	100%; 1325 / 1325
Sequence identity	100%
Num Substitutions	0
E-value	0

Show MGE alignment

Contig: 119assembly_contig_63 length 2383 coverage 163.2 normalized_cov 1.19

Resistance results

Gene name	Phenotype	Accession	Position in contig	Coverage	Identity
tet(B)	tetracycline, minocycline, doxycycline	AP000342	1088-2293	100%	100%

Contig: 119assembly_contig_53 length 7500 coverage 185.0 normalized_cov 1.35

Resistance results

Gene name	Phenotype	Accession	Position in contig	Coverage	Identity
qacE	ethidium bromide, cetylpyridinium chloride, benzylkonium chloride, chlorhexidine	X68232	4025-4306	84.68468468468468%	100%
sul1	sulfamethoxazole	U12338	3126-3965	100%	100%
ARR-2	rifampicin	HQ141279	6078-6530	100%	100%
cmlA1	chloramphenicol	M64556	4498-5757	100%	99.68253968253968%

ISEc28

Family	IS5
Group	IS903
Type	Insertion sequence
Reference db	isfinder
Accession	FJ187822
Position in contig	51-947
Strand	reverse
Alignment coverage	100%; 897 / 897
Sequence identity	99.89%
Num Substitutions	1
E-value	0

Show MGE alignment

Contig: 119assembly_contig_13 length 120400 coverage 125.5 normalized_cov 0.92

Resistance results

Gene name	Phenotype	Accession	Position in contig	Coverage	Identity
blaOXA-64	unknown beta-lactam	AY750907	89510-90334	100%	100%

Contig: 119assembly_contig_62 length 2443 coverage 225.7 normalized_cov 1.65

Resistance results

Gene name	Phenotype	Accession	Position in contig	Coverage	Identity
blaOXA-23	meropenem, imipenem	AY795964	34-855	100%	100%

Contig: 119assembly_contig_10 length 140413 coverage 132.0 normalized_cov 0.96

Resistance results

Gene name	Phenotype	Accession	Position in contig	Coverage	Identity
blaADC-25	unknown beta-lactam	EF016355	85536-86687	100%	97.22222222222221%

Contig: 119assembly_contig_77 length 1164 coverage 230.6 normalized_cov 1.68

Resistance results

Gene name	Phenotype	Accession	Position in contig	Coverage	Identity
sul2	sulfamethoxazole	AY034138	1143-328	100%	100%

Contig: 119assembly_contig_60 length 3268 coverage 114.9 normalized_cov 0.84

Resistance results

Gene name	Phenotype	Accession	Position in contig	Coverage	Identity
blaNDM-1	amoxicillin+clavulanic acid, temocillin, piperacillin, cefepime, ertapenem, ampicillin+clavulanic acid, meropenem, piperacillin+tazobactam, ampicillin, imipenem, cefixime, cefotaxime, cefoxitin, amoxicillin, ceftazidime	FN396876	1-793	97.539975399754%	100%

Contig: 119assembly_contig_30 length 46262 coverage 184.3 normalized_cov 1.34

IS1007

Family	IS6
Type	Insertion sequence
Reference db	isfinder
Accession	AJ250860
Position in contig	33693-34510
Strand	reverse
Alignment coverage	99.63%; 818 / 819
Sequence identity	95.37%
Num Substitutions	35
E-value	0

Show MGE alignment

cn_10921_IS1007

Family	IS6
Type	Composite transposon
Reference db	isfinder
Accession	AJ250860
Position in contig	33692-44613
Strand	reverse
Prediction	Putative MGE
Alignment coverage of flanking MGEs	99.63%
Sequence identity of flanking MGEs	95.37%
Num Substitutions in flanking MGEs	35
E-value	0

Show MGE alignment

Contig: 119assembly_contig_35 length 39839 coverage 127.0 normalized_cov 0.93

ISAba33

Family	IS4
Group	IS10
Type	Insertion sequence
Reference db	isfinder
Accession	KT852972
Position in contig	38619-39777
Strand	forward
Truncation description	**5p truncation:** 21 nt
Alignment coverage	98.22%; 1159 / 1180
Sequence identity	98.19%
Num Substitutions	21
E-value	0

Show MGE alignment

ISAba10

Family	IS5
Group	IS903
Type	Insertion sequence
Reference db	isfinder
Accession	GQ379223
Position in contig	37022-38043

Strand	reverse
Alignment coverage	99.9%; 1022 / 1023
Sequence identity	98.92%
Num Substitutions	10
E-value	0

Show MGE alignment

Contig: 119assembly_contig_49 length 9892 coverage 192.1 normalized_cov 1.40

ISAba14

Synonyms	ISAba35
Family	IS3
Group	IS150
Type	Insertion sequence
Reference db	isfinder
Accession	CP001921
Position in contig	5646-6927
Strand	forward
Alignment coverage	99.84%; 1282 / 1282
Sequence identity	95.56%
Num Substitutions	55
E-value	0

Show MGE alignment

ISAba37

Family	IS5
Group	IS1031
Type	Insertion sequence
Reference db	isfinder
Accession	KU744946
Position in contig	7714-8585
Strand	reverse
Alignment coverage	100%; 872 / 872
Sequence identity	100%
Num Substitutions	0
E-value	0

Show MGE alignment

Contig: 119assembly_contig_64 length 1984 coverage 298.9 normalized_cov 2.18

ISVsa3

Synonyms	ISVs3
Family	IS91
Type	Insertion sequence
Reference db	isfinder
Accession	AJ289135
Position in contig	28-1004
Strand	forward
Alignment coverage	100%; 977 / 977
Sequence identity	100%
Num Substitutions	0
E-value	0

Show MGE alignment

Contig: 119assembly_contig_70 length 1309 coverage 607.8 normalized_cov 4.43

ISAba34

Family	IS3
Group	IS51
Type	Insertion sequence
Reference db	isfinder
Accession	KU744946
Position in contig	1-1309
Strand	reverse
Alignment coverage	100%; 1309 / 1309
Sequence identity	100%
Num Substitutions	0
E-value	0

Show MGE alignment

Contig: 119assembly_contig_72 length 1284 coverage 482.2 normalized_cov 3.52

ISAba14

Synonyms	ISAba35
Family	IS3
Group	IS150
Type	Insertion sequence
Reference db	isfinder
Accession	CP001921
Position in contig	1-1282
Strand	forward
Alignment coverage	100%; 1282 / 1282
Sequence identity	99.84%
Num Substitutions	2
E-value	0

Show MGE alignment

Contig: 119assembly_contig_75 length 1186 coverage 634.3 normalized_cov 4.63

ISAca1

Family	IS3
Group	IS407
Type	Insertion sequence
Reference db	isfinder
Accession	AF121266
Position in contig	1-1186
Strand	forward
Alignment coverage	99.83%; 1186 / 1186
Sequence identity	90.82%
Num Substitutions	107
E-value	0

Show MGE alignment

Contig: 119assembly_contig_83 length 821 coverage 397.2 normalized_cov 2.90

IS1008

Family	IS6
Type	Insertion sequence
Reference db	isfinder
Accession	AJ251307
Position in contig	2-821
Strand	forward
Alignment coverage	100%; 820 / 820
Sequence identity	100%
Num Substitutions	0
E-value	0

Show MGE alignment

Contig: 119assembly_contig_84 length 820 coverage 325.8 normalized_cov 2.38

IS26

Synonyms	IS160,IS26L,IS26R,IS6,IS140,IS46
Family	IS6
Type	Insertion sequence
Reference db	isfinder
Accession	X00011
Position in contig	1-820
Strand	forward
Alignment coverage	100%; 820 / 820
Sequence identity	100%
Num Substitutions	0
E-value	0

Show MGE alignment

CITATIONS

For publication of results, please cite:

- Detection of mobile genetic elements associated with antibiotic resistance in Salmonella enterica using a newly developed web tool: MobileElementFinder.
  Johansson, Markus H K and Bortolaia, Valeria and Tansirichaiya, Supathep and Aarestrup, Frank M and Roberts, Adam P and Petersen, Thomas N.
  Journal of Antimicrobial Chemotherapy. 2020 Oct 3.
  PMID: 33009809 doi: 10.1093/jac/dkaa390

Copyright DTU 2011 / All rights reserved

Center for Genomic Epidemiology, DTU, Kemitorvet, Building 204, 2800 Kgs. Lyngby, Denmark
Contact: Vibeke Dybdahl Hammer, Telephone: +45 3588 6420, E-mail: vdha@food.dtu.dk

Funded by: The Danish Council for Strategic Research
Last modified May 22, 2012 11:08:01 GMT

@article{Johansson2020, abstract = {Antimicrobial resistance (AMR) in clinically relevant bacteria is a growing threat to public health globally. In these bacteria, antimicrobial resistance genes are often associated with mobile genetic elements (MGEs), which promote their mobility, enabling them to rapidly spread throughout a bacterial community.The tool MobileElementFinder was developed to enable rapid detection of MGEs and their genetic context in assembled sequence data. MGEs are detected based on sequence similarity to a database of 4452 known elements augmented with annotation of resistance genes, virulence factors and detection of plasmids.MobileElementFinder was applied to analyse the mobilome of 1725 sequenced Salmonella enterica isolates of animal origin from Denmark, Germany and the USA. We found that the MGEs were seemingly conserved according to multilocus ST and not restricted to either the host or the country of origin. Moreover, we identified putative translocatable units for specific aminoglycoside, sulphonamide and tetracycline genes. Several putative composite transposons were predicted that could mobilize, among others, AMR, metal resistance and phosphodiesterase genes associated with macrophage survivability. This is, to our knowledge, the first time the phosphodiesterase-like pdeL has been found to be potentially mobilized into S. enterica.MobileElementFinder is a powerful tool to study the epidemiology of MGEs in a large number of genome sequences and to determine the potential for genomic plasticity of bacteria. This web service provides a convenient method of detecting MGEs in assembled sequence data. MobileElementFinder can be accessed at https://cge.cbs.dtu.dk/services/MobileElementFinder/.}, author = {}, doi = {10.1093/jac/dkaa390}, issn = {0305-7453}, journal = {Journal of Antimicrobial Chemotherapy}, month = {oct}, title = {{}}, url = {https://doi.org/10.1093/jac/dkaa390}, year = {2020} }
