# Supplementary material for: Whole-genome sequencing of two multidrug-resistant acinetobacter baumannii strains isolated from a neonatal intensive care unit in Egypt: a prospective cross-sectional study
Source: BMC Microbiol. 2024 Sep 21;24:362. doi: 10.1186/s12866-024-03482-3 (PMC11415996; doi:10.1186/s12866-024-03482-3)
Supplement: Supplementary file 2 — Supplementary Material 2. [file 12866_2024_3482_MOESM2_ESM.pdf]

4930670.cgebase.food.dtu.dk 4930671.cgebase.food.dtu.dk 4930672.cgebase.food.dtu.dk 4930673.cgebase.food.dtu.dk 4930674.cgebase.food.dtu.dk

Contig: 123assemby_contig_90 length 6078 coverage 9368.8 normalized_cov 56.80

Resistance results

Gene name	Phenotype	Accession	Position in contig	Coverage	Identity
ant(2")-Ia	tobramycin, gentamicin	AY139599	5590-6078	91.57303370786516%	98.97750511247445%
ant(2")-Ia	tobramycin, gentamicin	DQ176450	5590-6078	91.57303370786516%	98.97750511247445%

Contig: 123assemby_contig_57 length 18942 coverage 108.1 normalized_cov 0.66

Resistance results

Gene name	Phenotype	Accession	Position in contig	Coverage	Identity
aph(3")-Ib	streptomycin	AF321550	15206-16009	100%	99.87562189054727%
aph(3")-Ib	streptomycin	AF024602	15207-16009	99.87562189054727%	100%
aph(6)-Id	streptomycin	M28829	16009-16845	100%	100%
aph(3")-Ib	streptomycin	AF321551	15206-16009	100%	99.87562189054727%
aph(3")-Ib	streptomycin	AF313472	15206-16009	100%	99.87562189054727%

ISVsa3

Synonyms	ISVs3
Family	IS91
Type	Insertion sequence
Reference db	isfinder
Accession	AJ289135
Position in contig	17930-18906
Strand	reverse
Alignment coverage	100%; 977 / 977
Sequence identity	100%
Num Substitutions	0
E-value	0

Show MGE alignment

Contig: 123assemby_contig_117 length 898 coverage 104.4 normalized_cov 0.63

Resistance results

Gene name	Phenotype	Accession	Position in contig	Coverage	Identity
aph(3')-VIa	paromomycin, butirosin, neomycin, amikacin, kanamycin, gentamicin, ribostamycin	X07753	873-94	100%	99.87179487179488%

Contig: 123assemby_contig_36 length 39343 coverage 142.5 normalized_cov 0.86

Resistance results

Gene name	Phenotype	Accession	Position in contig	Coverage	Identity
blaADC-25	unknown beta-lactam	EF016355	38278-39343	92.53472222222221%	96.62288930581614%

Contig: 123assemby_contig_89 length 7438 coverage 115.0 normalized_cov 0.70

Resistance results

Gene name	Phenotype	Accession	Position in contig	Coverage	Identity
msr(E)	virginiamycin s, quinupristin, pristinamycin ia, erythromycin, azithromycin	FR751518	2663-4138	100%	100%
armA	isepamicin, netilmicin, tobramycin, amikacin, gentamicin	AY220558	6437-7210	100%	100%
mph(E)	erythromycin	DQ839391	1723-2607	100%	100%

ISEc29

Family	IS4
Group	IS10
Type	Insertion sequence
Reference db	isfinder
Accession	FJ187822
Position in contig	4503-5827
Strand	reverse
Alignment coverage	100%; 1325 / 1325
Sequence identity	100%
Num Substitutions	0
E-value	0

Show MGE alignment

Contig: 123assemby_contig_63 length 14531 coverage 3662.9 normalized_cov 22.21

Resistance results

Gene name	Phenotype	Accession	Position in contig	Coverage	Identity
tet(39)	doxycycline, tetracycline	KT346360	7782-8903	100%	100%

Contig: 123assemby_contig_67 length 13477 coverage 185.4 normalized_cov 1.12

Resistance results

Gene name	Phenotype	Accession	Position in contig	Coverage	Identity
sul1	sulfamethoxazole	U12338	9077-9916	100%	100%
blaPER-7	cefepime, amoxicillin+clavulanic acid, ticarcillin+clavulanic acid, ticarcillin, ampicillin+clavulanic acid, ceftazidime, piperacillin+tazobactam, aztreonam, ampicillin, piperacillin, cefoxitin, cefotaxime, amoxicillin	HQ713678	5880-6806	100%	100%
ARR-2	rifampicin	HQ141279	12029-12481	100%	100%

Gene name	Phenotype	Accession	Position in contig	Coverage	Identity
cmlA1	chloramphenicol	M64556	10449-11708	100%	99.68253968253968%
sul1	sulfamethoxazole	U12338	3126-3965	100%	100%
qacE	cetylpyridinium chloride, chlorhexidine, benzylkonium chloride, ethidium bromide	X68232	10257-9976	84.68468468468468%	100%

ISEc28

Family	IS5
Group	IS903
Type	Insertion sequence
Reference db	isfinder
Accession	FJ187822
Position in contig	51-947
Strand	reverse
Alignment coverage	100%; 897 / 897
Sequence identity	99.89%
Num Substitutions	1
E-value	0

Show MGE alignment

Contig: 123assemby_contig_68 length 13415 coverage 140.5 normalized_cov 0.85

Resistance results

Gene name	Phenotype	Accession	Position in contig	Coverage	Identity
blaOXA-68	unknown beta-lactam	AY750910	6363-7187	100%	100%

Contig: 123assemby_contig_114 length 1036 coverage 130.7 normalized_cov 0.79

Resistance results

Gene name	Phenotype	Accession	Position in contig	Coverage	Identity
tet(B)	minocycline, doxycycline, tetracycline	AP000342	1-946	78.44112769485903%	100%

Contig: 123assemby_contig_81 length 9013 coverage 142.5 normalized_cov 0.86

Resistance results

Gene name	Phenotype	Accession	Position in contig	Coverage	Identity
blaOXA-23	meropenem, imipenem	AY795964	28-849	100%	100%

Contig: 123assemby_contig_108 length 1361 coverage 218.4 normalized_cov 1.32

Resistance results

Gene name	Phenotype	Accession	Position in contig	Coverage	Identity
sul2	sulfamethoxazole	AY034138	22-837	100%	100%

Contig: 123assemby_contig_31 length 48136 coverage 173.4 normalized_cov 1.05

IS1007

Family	IS6
Type	Insertion sequence
Reference db	isfinder
Accession	AJ250860
Position in contig	33693-34510
Strand	reverse
Alignment coverage	99.63%; 818 / 819
Sequence identity	95.37%
Num Substitutions	35
E-value	0

Show MGE alignment

---

cn_10921_IS1007

Family	IS6
Type	Composite transposon
Reference db	isfinder
Accession	AJ250860
Position in contig	33692-44613
Strand	reverse
Prediction	Putative MGE
Alignment coverage of flanking MGEs	99.63%
Sequence identity of flanking MGEs	95.37%
Num Substitutions in flanking MGEs	35
E-value	0

Show MGE alignment

Contig: 123assemby_contig_77 length 9952 coverage 174.6 normalized_cov 1.06

ISAba14

Synonyms	ISAba35
Family	IS3
Group	IS150
Type	Insertion sequence
Reference db	isfinder
Accession	CP001921
Position in contig	2958-4239
Strand	reverse
Alignment coverage	99.84%; 1282 / 1282
Sequence identity	95.56%
Num Substitutions	55
E-value	0

Show MGE alignment

ISAba37

Family	IS5
Group	IS1031
Type	Insertion sequence
Reference db	isfinder
Accession	KU744946
Position in contig	1300-2171
Strand	forward
Alignment coverage	100%; 872 / 872

Sequence identity	100%
Num Substitutions	0
E-value	0

Show MGE alignment

Contig: 123assemby_contig_99 length 3838 coverage 163.4 normalized_cov 0.99

ISAba34

Family	IS3
Group	IS51
Type	Insertion sequence
Reference db	isfinder
Accession	KU744946
Position in contig	1135-2434
Strand	reverse
Alignment coverage	99.31%; 1300 / 1309
Sequence identity	99.31%
Num Substitutions	0
E-value	0

Show MGE alignment

Contig: 123assemby_contig_112 length 1177 coverage 2022.3 normalized_cov 12.26

ISAca1

Family	IS3
Group	IS407
Type	Insertion sequence
Reference db	isfinder
Accession	AF121266
Position in contig	1-1177
Strand	forward
Alignment coverage	99.07%; 1177 / 1186
Sequence identity	90.75%
Num Substitutions	107
E-value	0

Show MGE alignment

Contig: 123assemby_contig_121 length 820 coverage 513.1 normalized_cov 3.11

IS1008

Family	IS6
Type	Insertion sequence
Reference db	isfinder
Accession	AJ251307
Position in contig	1-820
Strand	forward
Alignment coverage	100%; 820 / 820
Sequence identity	100%
Num Substitutions	0
E-value	0

Show MGE alignment

CITATIONS

For publication of results, please cite:

- Detection of mobile genetic elements associated with antibiotic resistance in Salmonella enterica using a newly developed web tool: MobileElementFinder.
  Johansson, Markus H K and Bortolaia, Valeria and Tansirichaiya, Supathep and Aarestrup, Frank M and Roberts, Adam P and Petersen, Thomas N.
  Journal of Antimicrobial Chemotherapy. 2020 Oct 3.
  PMID: 33009809 doi: 10.1093/jac/dkaa390

Support Scientific problems Technical problems

Copyright DTU 2011 / All rights reserved

Center for Genomic Epidemiology, DTU, Kemitorvet, Building 204, 2800 Kgs. Lyngby, Denmark
Contact: Vibeke Dybdahl Hammer, Telephone: +45 3588 6420, E-mail: vdha@food.dtu.dk

Funded by: The Danish Council for Strategic Research
Last modified May 22, 2012 11:08:01 GMT

@article{Johansson2020, abstract = {Antimicrobial resistance (AMR) in clinically relevant bacteria is a growing threat to public health globally. In these bacteria, antimicrobial resistance genes are often associated with mobile genetic elements (MGEs), which promote their mobility, enabling them to rapidly spread throughout a bacterial community.The tool MobileElementFinder was developed to enable rapid detection of MGEs and their genetic context in assembled sequence data. MGEs are detected based on sequence similarity to a database of 4452 known elements augmented with annotation of resistance genes, virulence factors and detection of plasmids.MobileElementFinder was applied to analyse the mobilome of 1725 sequenced Salmonella enterica isolates of animal origin from Denmark, Germany and the USA. We found that the MGEs were seemingly conserved according to multilocus ST and not restricted to either the host or the country of origin. Moreover, we identified putative translocatable units for specific aminoglycoside, sulphonamide and tetracycline genes. Several putative composite transposons were predicted that could mobilize, among others, AMR, metal resistance and phosphodiesterase genes associated with macrophage survivability. This is, to our knowledge, the first time the phosphodiesterase-like pdeL has been found to be potentially mobilized into S. enterica.MobileElementFinder is a powerful tool to study the epidemiology of MGEs in a large number of genome sequences and to determine the potential for genomic plasticity of bacteria. This web service provides a convenient method of detecting MGEs in assembled sequence data. MobileElementFinder can be accessed at https://cge.cbs.dtu.dk/services/MobileElementFinder/.}, author = {}, doi = {10.1093/jac/dkaa390}, issn = {0305-7453}, journal = {Journal of Antimicrobial Chemotherapy}, month = {oct}, title = {{}}, url = {https://doi.org/10.1093/jac/dkaa390}, year = {2020} }
